# Retinal exams requested at Primary Care Unit: indications, results and alternative strategies of evaluation

**DOI:** 10.31744/einstein_journal/2020GS4913

**Published:** 2019-09-11

**Authors:** Fernando Korn Malerbi, Adriano Biondi Monteiro Carneiro, Marcelo Katz, Claudio Luiz Lottenberg

**Affiliations:** 1 Hospital Israelita Albert Einstein, São Paulo, SP, Brazil.

**Keywords:** Telemedicine, Diabetic retinopathy, Glaucoma, Cost efficiency analysis, Health Centers, Primary Health Care

## Abstract

**Objective:**

To evaluate indications, results and strategy of retinal exams requested at Primary Care Units.

**Methods:**

A retrospective study that analyzed the indications and results of retinal exams, in the modalities clinical dilated fundus exams and color fundus photographs. In the following situations, patients were considered eligible for color fundus photographs if visual acuity was normal and ocular symptoms were absent: *diabetes mellitus* and/or hypertension, in use of drugs with potential retinal toxicity, diagnosis or suspicion of glaucoma, stable and asymptomatic retinopathies, except myopia greater than -3.00 diopters.

**Results:**

A total of 1,729 patients were evaluated (66% female, age 63.5±15.5 years), and 1,190 underwent clinical dilated fundus exam and 539 underwent color fundus photographs. Diabetes was present in 32.2%. The main indications were diabetes (23.7%) and glaucoma evaluation (23.5%). In 3.4% of patients there was no apparent indication. The main results were a large cup/disc ratio (30.7%) and diabetic retinopathy (13.2%). Exam was normal in 9.6%, detected peripheral changes in 7% and could not be performed in 1%. Considering patients eligible for fundus photographs (22.4%), more than half underwent clinical dilated fundus exams.

**Conclusion:**

Regarding exam modality, there were no important differences in the distribution of indications or diagnosis. Color fundus photograph is compatible with telemedicine and more cost-effective, and could be considered the strategy of choice in some scenarios. Since there are no clear guidelines for retinal exams indications or the modality of choice, this study may contribute to such standardization, in order to optimize public health resources.

## INTRODUCTION

Retinal tests may be requested in various clinical situations, such as in the investigation of low visual acuity, or ocular or systemic conditions with the potential of affecting the retina or the optic nerve. Retinal diseases are the most common causes of blindness in adults of urban populations in Brazil. ^1^ Diabetic retinopathy (DR) is the primary cause of blindness in the economically active population in developed countries. ^2^ Tracking DR, as well as the periodic retinal examination in diabetic patients, is part of the international recommendations for ocular care in diabetes. ^3^ In Brazil, besides DR, age-related macular degeneration (AMD), and retinal detachment are important retinal causes of blindness.^1^ The primary open-angle glaucoma (POAG) is a disease that can lead to optic nerve damage, a structure that is also evaluated during examination of the posterior ocular segment.

The primary methods to evaluate the retina are clinical dilated fundus exam (DFE), performed by a specialist and digital photography of the retina, called color fundus photographs (CFP), which can be obtained by a non-medical professional and later interpreted by a specialist. Both modes are effective for detecting modifications in the retina and optic nerve, and CFP can be integrated to telemedicine protocols, a combination that offers a good cost-effective profile,^4 - 7^ besides allowing expanded access to diagnosis when there is an imbalance between offer and demand for specialists.^8 , 9^ Currently, in the Brazilian public health system, there are no precise guidelines as to the type of retinal examination that should be requested.

## OBJECTIVE

To evaluate the indications, results, and strategy of retinal examinations requested for patients of the Primary Care Units.

## METHODS

This is a retrospective study, based on the analysis of medical records of patients examined at the Ophthalmology Diagnosis Center (CDOF - *Centro Diagnóstico de Oftalmologia* ) of the *Instituto Israelita de Responsabilidade Social Albert Einstein* (IIRS). Tests performed within the period of approximately 10 years, between October 31, 2007 and June 5, 2017, were analyzed.

All tests were requested by the same medical staff composed of 21 ophthalmologists of *Hospital Israelita Albert Einstein* (HIAE), who worked at Primary Care Units, situated in peripheral zones of the city of São Paulo (SP). The care delivered at the Primary Care Units and the performance of tests at the CDOF were part of the partnership established between the IIRS and the City Administration of São Paulo.

Dilated fundus exam was conducted by two ophthalmologists specialized in retinal diseases, with indirect binocular ophthalmoscopy complemented by biomicroscopic fundoscopy with a slit lamp.

Called CFP assessments were performed by ophthalmic technicians on Zeiss FF 450 equipment (Carl Zeiss Meditec AG, Jena, Germany), with an angle of 50°, and documentation of the posterior pole and periphery. Such images were interpreted by the same two physicians specialized in retinal diseases.

The following clinical and demographic data were analyzed: sex, age, corrected visual acuity, presence of diabetes (self-reported), main indication of the retinal examination, and results of this test. Indication of the test was based on the ocular and/or systemic history, complaint and on visual acuity; in cases in which there was more than one complaint or medical condition, we considered the condition that posed more risk to vision. For patients who underwent more than one examination during that period, information was collected as to the first examination performed in the period.

For analysis of the indication of the retinal examination, the following clinical conditions were categorized ( Table 1 ): late postoperative period of eye surgery; myopia greater than -3.00 diopters in at least one of the eyes; investigation of systemic diseases with possible involvement of the posterior ocular segment; low visual acuity to be determined, or patient presenting with useful vision in one eye only (“single eye”); preoperative examination of cataract surgery or capsulotomy; use of oral medication with potential retinal toxicity; *diabetes mellitus* ; diagnosis or suspicion of POAG; hypertension; history of ocular trauma; personal or family ophthalmologic history; and complaint or clinical picture consistent with another ocular disease that possibly affects the posterior segment (not mentioned in the categories above). Also categorized were the patients who did not present with any evident indication of retinal examination (“routine evaluation”; included in this category were patients in the late postoperative period of phakectomy with no complications) and those for whom there were no data appropriately filled in on the medical record.

**Table 1:** Primary indications for retinal examinations, distributions per modality, and distribution of examinations with normal results

Indications	Total	Dilated fundus exam	Color fundus photography	p value^†^	Normal
	n (%)	(%)*	(%)*		n (%)
Late postoperative period of ocular surgery^‡^	66 (3.8)	74.2	25.7	0.333	0 (0)
Myopia greater than -3.00 diopters	123 (7.1)	84.5	15.4	0^†^	4 (2.4)
Investigation of systemic diseases§	67 (3.9)	79.1	20.9	0.064	12 (7.2)
Low visual acuity to be determined/”single eye”^¶^	121 (7)	81	19	0.003^†^	10 (6)
History of retinal disease^||^	280 (16.2)	71.1	28.9	0.376	20 (12)
Preoperative evaluation^#^	36 (2.1)	66.7	33.3	0.777	3 (1.8)
History of ocular trauma	14 (0.8)	57.1	42.8	0.343	3 (1.8)
Retinal toxicity**	25 (1.4)	68	32	0.928	7 (4.2)
Diabetes	409 (23.8)	71.6	28.3	0.16	57 (34.3)
Glaucoma^††^	406 (23.6)	49.7	50.2	0^†^	17 (10.2)
Hypertension	114 (6.6)	80.7	19.2	0.005^†^	11 (6.6)
No apparent indication	58 (3.4)	72.4	27.5	0.548	22 (13.2)
Total	1,719 (100)				166 (100)

* Dilated fundus exams added to color fundus photographs total up 100% in each category; ^†^ significant differences (p<0.05); ^‡^ except late postoperative period of phakectomy with no complications; ^§^ with possible involvement of the posterior ocular segment, except for diabetes and hypertension; ^¶^ patient presenting with useful vision in one eye only; ^||^ personal or family history, complaint or clinical picture consistent with ocular disease ocular with possible involvement of the posterior segment, except for diagnosis or investigation of primary open-angle glaucoma or high myopia; ^#^ phakectomy or capsulotomy; ** use of oral medication with potential retinal toxicity; ^††^ diagnosis or investigation of primary open-angle glaucoma.

The study was approved by the Ethics Committee of the organization, under no. 2.338.968. CAAE: 77215417.0.0000.0071.

For analysis of indication of the study strategy, the following situations were considered eligible for evaluation by CFP, in cases of good visual acuity (vision better than or equal to 20/30 in the worst eye) ^10^ and in the absence of symptoms (tracking): patients with *diabetes mellitus* ; hypertensive; patients on oral medication with potential retinal toxicity; with diagnosis or suspicion of POAG; and with stable and asymptomatic retinopathies, except for myopia greater than -3.00 diopters. ^11^ Such criteria are not a part of the consensus and were proposed for the conduction of this study.

As to analysis of the examination result, the evaluation took into consideration both eyes of each patient; when there was more than one diagnosis, the primary retinal modification was considered − the one that poses more risk to vision. Besides the primary diagnosis, the presence of DR and of the increase in cup/disc ratio was individually assessed.

For the statistical analysis, the IBM Statistical Package for the Social Sciences (SPSS) for Windows, version 20 (IBM Corp, Armonk, NY, USA) software was used. The continuous variables are described as mean and standard deviation, and the categorical variables, as absolute and relative frequencies. To compare continuous variables, the non-paired Student’s *t* test was used, and the χ^2^ test was employed to compare the categorical variables. The p value <0.05 was considered statistically significant.

## RESULTS

**Figure 1:**
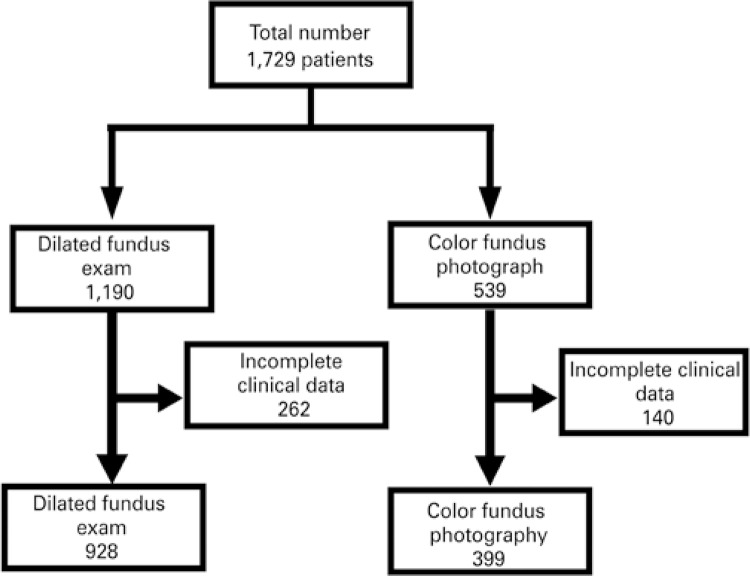
Patients and modalities of examinations

A total of 1,729 patients were evaluated; of these, 1,190 underwent DFE and 539, CFP. Figure 1 shows the total number of patients undergoing each exam, who presented with complete clinical data for analysis. Mean age of patients was 63.5±15.5 years, and 66% were female. The diagnosis of diabetes was present in 32.2% of patients.

Of the 1,729 patients, 10 (0.6%; 9 undergoing DFE, and 1 CFP) in whom it was not possible to evaluate the indication of the examination, were excluded. Fifty-eight (3.4%) patients had no apparent indication for the retinal exams as per the criteria numbered above; 42 patients were submitted to DFE and 16 to CFP. Table 1 also shows the reasons for indicating the exam in cases with a normal result, which occurred in 166 patients (9.6% of total), of which, 115 DFE (9.6% of total DFE) and 51 CFP (9.5% of the total CFP).

As to the modality of examination, 386 patients (22.4%) presented with an indication for retinal examination and were eligible for the CFP modality. Of all patients eligible for CFP, 229 (59.3%) were submitted to DFE, which corresponded to 13.3% of the overall total. The reasons for requesting examinations in this group of patients were follow-up of stable retinopathy (AMD, optic neuritis, and choroid nevus) in 16 patients (6.9%); use of oral medication with potential retinal toxicity in 8 patients (3.5%); diagnosis of diabetes in 83 patients (36.2%); diagnosis or investigation of POAG in 86 patients (37.5%); diagnosis of hypertension in 36 patients (15.7%).

The primary diagnoses found in the retinal examinations are shown on table 2 , the most frequent of which are the increase in cup/disc ratio (30.7%) and DR (13.2%). Table 2 also displays the results of the group of 58 patients who had tests requested with no apparent indication. In 121 patients (115 DFE and 6 CFP), or 7% of total number of exams, the retinal evaluation detected peripheral modifications in the forms of peripheral degeneration, rupture, or retinal break, or yet, retinal detachment. In 18 patients (1%), the evaluation was made impossible due to opacity of the media (10, or 0.8%, of DFE and 8, or 1.5%, CFP).

**Table 2:** Main diagnoses, distributions per modality of examination, and distribution of tests requested with no apparent indication

Diagnosis	Total	Dilated fundus exam	Color fundus photography	Examination with no apparent indication
	n (%)	(%)	(%)	(%)
Large cup/disc ratio	401 (23.2)	20.3	29.5	2 (3.4)
Peripheral modifications	99 (5.7)	8.2	0.4	7 (12.1)
AMD	110 (6.4)	5.9	7.4	4 (6.9)
Retinal detachment	25 (1.4)	1.8	0.7	0 (0)
Retinal dystrophies	34 (2)	1.3	3.3	2 (3.4)
Impossible	18 (1)	0.8	1.5	0 (0)
Toxic maculopathy	8 (0.5)	0.7	0	1 (1.7)
Degenerative myopia	93 (5.4)	6.6	2.8	0 (0)
Choroidal nevus	45 (2.6)	2.4	3.2	1 (1.7)
Normal	170 (9.8)	9.9	9.6	22 (37.9)
Retinal vascular occlusion	55 (3.2)	3.1	3.3	1 (1.7)
Diabetic retinopathy	221 (12.8)	13.1	12.1	0 (0)
Hypertensive retinopathy	122 (7.1)	8.5	3.9	2 (3.4)
Miscellaneous^*^	275 (15.9)	17.3	12.8	15 (25.8)
No data	53 (3.1)	0.2	9.5	1 (1.7)
Total	1.729 (100)	100	100	58 (100)

^*^ Includes: non-glaucomatous modifications of the optic nerve, pigmented modifications not associated with dystrophies, macular hole, congenital modifications, other maculopathy not classified in the items above, other retinal vascular diseases, epiretininal membrane, posterior uveitis, modification in the foveal reflex, choroidal folds, choroid drusen not associated with the age-related macular degeneration, posterior vitreous detachment, myelin fibers, asteroid hyalosis, phthisis bulbi, late postoperative period of vitreoretinal surgery, degenerative retinoschisis. AMD: age-related macular degeneration.

In the present study, the primary indications for performing the retinal examination for patients evaluated in a Primary Healthcare Unit were diabetes (23.7%) and investigation of glaucoma (23.5%); in diabetic individuals, the exam was normal in 34.3% of cases. The main diagnoses were large cup/disc ratio (23.2%) and DR (12.8%); in patients with a normal result, the main indication for the examination was the presence of diabetes (34.3%). It was considered that in only 3.4% of cases, the test was requested with no apparent indication. The DFE was the most frequently performed examination (68.8%); nevertheless, considering all patients eligible for use of the CFP modality, approximately 60% of these were submitted to DFE.

## DISCUSSION

Currently, there is no consensus as to the need for performing the retinal examination under mydriasis in individuals not presenting with signs or symptoms of diseases of the posterior ocular segment, or who have no risk factors for the development of such diseases, since conditions detected in routine examinations under pupil dilation and that generate changes in management or prevention of an outcome are rare.^11 - 13^ As examples, we can cite the detection of choroid nevus as an exam finding and its low risk of malignant transformation, ^14^ and modifications on the periphery of the retina not accompanied by symptoms, which generally are benign and do not demand treatment. ^15^ In this study, 121 patients (17%) presented with peripheral retinal modifications (115 submitted to DFE and 6 to CFP).

The American Academy of Ophthalmology (AAO) recommends that a complete ophthalmologic examination be done in 40-year-old individuals, but does not specifically mention pupil dilation. ^16^ The *Conselho Brasileiro de Oftalmologia* (CBO) [Brazilian Ophthalmology Council] drew up a protocol recommending the performance of DFE in the following situations: preoperative period of cataract surgery or of refractive surgery; acute or chronic entoptic phenomena; high myopia; family or personal history of retinal detachment, ocular trauma and hypoxic retinopathies (diabetes, thrombosis, Eales´ disease, and sickle cell anemia). ^17^

In clinical practice, however, tests for retinal examination are requested as part of the complementary ophthalmologic or clinical investigation in several other ocular or systemic conditions that potentially affect the posterior segment of the eye. Considering the two main strategies of retinal examination, DFE and CFP, there seems to be no clear indications as to the situations in which one or the other is most indicated; the choice should take into consideration some factors, such as availability of the exam, cost-effectiveness, and the specific clinical condition. For example, the investigation of predisposing lesions for retinal detachment in patients with myopia^18 , 19^ or in patients with symptoms of posterior vitreous detachment is a clear indication of DFE, which allows evaluation of the extreme periphery of the retina, contrary to the conventional CFP. ^13^ Such discussion is valid if we compare only conventional CFP devices, as there is equipment with ultra-wide fields that allow assessment of the extreme retinal periphery; nonetheless, at present, its cost is too high, hindering its current use in the public healthcare system. In the future, when such devices become more popular, it is possible that this discussion will become anachronic. ^20^

In most indications for the exam, there was no significant difference between the request of one or the other diagnostic modality, with the following exceptions: myopia greater than -3.00 diopters, investigation of low visual acuity and arterial hypertension, in which there was a significant predominance of DFE indication; and the investigation of glaucoma, in which the indication of CFP predominated. There were also no important differences in the distributions of diagnoses obtained by each modality.

Generally, both diagnostic methods for retinal evaluation studied show equivalent results and are strategically interchangeable, with some situations in which CFP can be considered the method of choice due to the cost-effectiveness profile,^4 - 8 , 20^ since it is compatible with telemedicine and does not require the presence of the specialist at the time of the examination. It has a high initial cost for equipment acquisition and structure assembly, which tends to be diluted posteriorly. In the situation studied here, the service already had the equipment, which conferred to this series a better cost-effective profile for the CFP strategy. Some studies carried out in Brazil have evaluated the adequacy of CFP associated with telemedicine for demand relief,^8 , 18 , 21^ especially in tracking DR within a scenario of a global epidemic of diabetes. ^22^ In the current study, CFP examinations were performed under mydriasis and achieved good technical quality; only 1.5% of the CFP tests did not allow reading due to poor image quality.

Additionally, evaluation by digital CFP enables interaction with artificial intelligence algorithms to help in tracking and in clinical decision-making, which shows a tendency for the future. ^23^ Also, the current imaging system, based on large equipment, should go through a big transformation with the possibility of obtaining images from portable CFP devices or smartphones *.*^24^

During the preparation of this article, Brazil was in the middle of a controversial situation regarding standardization of telemedicine, based on the resolution of the *Conselho Federal de Medicina* [Federal Medicine Council] published in February 2019, and revoked on February 22 (resolution 2.227/2018). ^25^ It should be emphasized that, if on the one hand, the use of telemedicine can represent the expanding access to diagnosis, on the other hand, retinal examination is just a part of the face-to-face ophthalmologic examination, which also includes history taking, refraction examination, biomicroscopy, extrinsic ocular mobility, and tonometry.

Among the strengths of this study, we can highlight the large number of patients examined at the same center, over a long period of approximately 10 years, and the evaluation of one of the scenarios that corresponds to the reality of our country. The presentation of these data may help in the planning of protocols with the objective of a better use of resources. Among the limitations of the study, we mention the possible biased choice of examination due to agenda restrictions, besides patients who had the exam requested and did not actually undergo the exam. Another bias focuses on the photographic documentation of patients with diagnosis or suspicion of glaucoma, since they had the availability of a special type of CFP, the stereoscopic photography of the papilla, which documents the optic nerve with greater magnification. Many patients of this category may have been submitted to this modality, and therefore the diagnosis of glaucoma or suspected glaucoma in the sample studied was underestimated. Even so, diagnosis or suspicion of glaucoma were two of the main indications for the examinations, and large cup/disc ratio was the primary diagnosis found. Finally, there are cases of patients who had both types of exam modalities requested, but only the first exam to be done was considered for the analysis of indications and results, adding one more bias in our results and conclusions.

## CONCLUSION

The data assessed in this study point to the fact that the primary indications for requests of the retinal exam were diabetes and the investigation of glaucoma, and the main results of the exams were large cup/disc ratio and diabetic retinopathy. In the absence of well-defined guidelines for the request of exams for retinal evaluation, and with no criteria for the choice of modality of the retinal exam, data revealed that, in daily clinical practice, the exam modality is generally chosen in an interchangeable manner. Considering that the different strategies studied have distinct profiles of cost-effectiveness, it is suggested to establish guidelines for indications of retinal examinations and the standardization of the modality most indicated in each situation, to optimize resources of the public healthcare system.
