# Supplementary material for: Retinal exams requested at Primary Care Unit: indications, results and alternative strategies of evaluation
Source: Einstein (Sao Paulo). 2019 Sep 11;18:eGS4913. doi: 10.31744/einstein_journal/2020GS4913 (PMC6905169; doi:10.31744/einstein_journal/2020GS4913)
Supplement: Translation

# Translation: Exames de retina solicitados em Unidades Básicas de Saúde: indicações, resultados e estratégias alternativas de avaliação

**Authors:** Fernando Korn Malerbi, Adriano Biondi Monteiro Carneiro, Marcelo Katz, Claudio Luiz Lottenberg

**Affiliations:** 1 Hospital Israelita Albert Einstein, São Paulo, SP, Brasil.

**Keywords:** Telemedicina, Retinopatia diabética, Glaucoma, Análise custo-eficiência, Centros de Saúde, Atenção Primária à Saúde

## Abstract

**Objetivo:**

Avaliar as indicações, os resultados e a estratégia de exames de retina solicitados em Unidades Básicas de Saúde.

**Métodos:**

Estudo retrospectivo no qual foram analisados as indicações e os resultados de exames de retina, nas modalidades mapeamento de retina e retinografia. Em casos de boa acuidade visual e sintomas oculares ausentes, foram considerados elegíveis para avaliação por retinografia: pacientes com diabetes mellitus e/ou hipertensão arterial sistêmica, em uso de medicação com potencial toxicidade retiniana, diagnóstico ou suspeita de glaucoma, e retinopatias estáveis e assintomáticas, exceto miopia maior que -3,00 dioptrias.

**Resultados:**

Foram avaliados 1.729 pacientes (66% do sexo feminino, idade 63,5±15,5 anos). Destes, 1.190 realizaram mapeamento de retina e 539 realizaram retinografia. Diabetes estava presente em 32,2%. As principais indicações para solicitação do exame foram diabetes (23,7%) e investigação de glaucoma (23,5%). Em 3,4%, não havia indicação aparente. Os principais resultados foram aumento da escavação papilar (30,7%) e retinopatia diabética (13,2%). O exame foi normal em 9,6%; detectou alterações periféricas em 7%; e sua realização foi impossível em 1%. Dos pacientes elegíveis para retinografia (22,4%), mais da metade foi submetida ao mapeamento de retina.

**Conclusão:**

Não houve diferenças importantes nas distribuições de indicações ou diagnósticos em relação à modalidade de exame. A retinografia, compatível com telemedicina e mais custo-efetiva, pode ser considerada a modalidade de escolha em determinadas situações. Na ausência de consenso quanto às indicações para a solicitação de exames da retina ou sua modalidade, este estudo pode contribuir para tal padronização, de modo a otimizar recursos do sistema público de saúde.

## INTRODUÇÃO

Exames de retina podem ser solicitados em diversas situações clínicas, como na investigação de baixa acuidade visual, ou de condições oculares ou sistêmicas com potencial de acometimento da retina ou do nervo óptico. Doenças da retina são a causa mais comum de cegueira em adultos em populações urbanas no Brasil.^1^ A retinopatia diabética (RD) é a principal causa de cegueira na população economicamente ativa nos países desenvolvidos.^2^ O rastreamento da RD, assim como o exame periódico da retina em portadores de diabetes, faz parte das recomendações internacionais para os cuidados oculares em diabetes.^3^ Além da RD, em nosso meio, a degeneração macular relacionada à idade (DMRI) e o descolamento de retina são importantes causas retinianas de cegueira.^1^ O glaucoma primário de ângulo aberto (GPAA) é uma doença que pode levar a danos no nervo óptico, estrutura que também é avaliada durante exame do segmento posterior ocular.

Os principais modos de se avaliar a retina são: o exame de mapeamento de retina (MR), realizado por especialista, e a fotografia digital da retina, denominada retinografia (RET), que pode ser obtida por profissional não médico e, posteriormente, interpretada pelo especialista. Ambos os modos são eficazes para a detecção de alterações da retina e do nervo óptico, sendo que a RET pode ser integrada a protocolos de telemedicina, combinação que apresenta bom perfil de custo-efetividade,^4 - 7^ além de permitir ampliação do acesso ao diagnóstico, quando há desequilíbrio entre a oferta e a demanda de especialistas.^8 , 9^ Atualmente, no sistema público de saúde brasileiro, não há diretrizes precisas em relação ao tipo de exame de retina que deve ser solicitado.

## OBJETIVO

Avaliar as indicações, os resultados e a estratégia de exame da retina solicitados para pacientes de Unidades Básicas de Saúde.

## MÉTODOS

Trata-se de estudo retrospectivo, realizado a partir da análise de prontuários de pacientes examinados no Centro Diagnóstico de Oftalmologia (CDOF) do Instituto Israelita de Responsabilidade Social Albert Einstein (IIRS). Foram analisados exames realizados no período de aproximadamente 10 anos, compreendidos entre 31 de outubro de 2007 e 5 de junho de 2017.

Todos os exames foram solicitados por um mesmo corpo clínico composto por 21 médicos oftalmologistas do Hospital Israelita Albert Einstein (HIAE), que atuavam em Unidades Básicas de Saúde, situadas em zonas periféricas da cidade de São Paulo (SP). O atendimento nas Unidades Básicas de Saúde e a realização de exames no CDOF faziam parte de parceria estabelecida entre o IIRS e a Prefeitura Municipal de São Paulo.

Os MR foram realizados por dois oftalmologistas especializados em doenças da retina, com oftalmoscopia binocular indireta complementada por biomicroscopia de fundo em lâmpada de fenda.

As RET foram realizadas por tecnólogos oftálmicos em equipamento Zeiss FF 450 (Carl Zeiss Meditec AG, Jena, Alemanha), com ângulo de 50°, e documentação de polo posterior e periferia. Tais imagens foram interpretadas pelos mesmos dois médicos especializados em doenças da retina.

Foram avaliados os seguintes dados clínicos e demográficos: sexo, idade, acuidade visual corrigida, presença de diabetes (autorreferida), principal indicação do exame de retina e resultados do exame de retina. A indicação do exame baseou-se no histórico ocular e/ou sistêmico, na queixa e na acuidade visual; nos casos em que existia mais de uma queixa ou condição clínica, foi considerada aquela que mais trazia risco à visão. Para pacientes que realizaram mais de um exame no período, foram colhidas as informações referentes ao primeiro exame realizado no período.

Para a análise da indicação do exame de retina, as seguintes situações clínicas foram categorizadas ( Tabela 1 ): pós-operatório tardio de cirurgia ocular; miopia em pelo menos um dos olhos maior que -3,00 dioptrias; investigação de doenças sistêmicas com possível acometimento do segmento posterior ocular; baixa acuidade visual a esclarecer ou paciente que apresentava visão útil em apenas um dos olhos (“olho único”); avaliação pré-operatória de cirurgia de catarata ou de capsulotomia; uso de medicação oral com potencial de toxicidade retiniana; *diabetes mellitus* ; diagnóstico ou suspeita de GPAA; hipertensão arterial sistêmica; antecedente de trauma ocular; antecedente oftalmológico pessoal ou familiar; e queixa ou quadro clínico compatíveis com outra doença ocular com possível acometimento do segmento posterior (não citada nas categorias acima). Também foram categorizados os pacientes que não apresentavam indicação evidente do exame da retina (“avaliação de rotina”; nesta categoria foram incluídos pacientes em pós-operatório tardio de facectomia sem intercorrências) e aqueles para os quais não existiam dados adequadamente preenchidos em prontuário.

**Tabela 1:** Principais indicações de exames de retina, distribuições por modalidade de exame e distribuições dos exames com resultado normal

Indicações	Total	Mapeamentos de retina	Retinografias	Valor de p^†^	Normais
	n (%)	(%)*	(%)*		n (%)
Pós-operatório tardio de cirurgia ocular^‡^	66 (3,8)	74,2	25,7	0,333	0 (0)
Miopia maior que -3,00 dioptrias	123 (7,1)	84,5	15,4	0^†^	4 (2,4)
Investigação de doenças sistêmicas^§^	67 (3,9)	79,1	20,9	0,064	12 (7,2)
Baixa visual a esclarecer/”olho único”^¶^	121 (7)	81	19	0,003^†^	10 (6)
Antecedente de doença retiniana^||^	280 (16,2)	71,1	28,9	0,376	20 (12)
Avaliação pré-operatória^#^	36 (2,1)	66,7	33,3	0,777	3 (1,8)
Antecedente de trauma ocular	14 (0,8)	57,1	42,8	0,343	3 (1,8)
Toxicidade retiniana**	25 (1,4)	68	32	0,928	7 (4,2)
Diabetes	409 (23,8)	71,6	28,3	0,16	57 (34,3)
Glaucoma^††^	406 (23,6)	49,7	50,2	0^†^	17 (10,2)
Hipertensão arterial sistêmica	114 (6,6)	80,7	19,2	0,005^†^	11 (6,6)
Sem indicação aparente	58 (3,4)	72,4	27,5	0,548	22 (13,2)
Total	1.719 (100)				166 (100)

* Mapeamentos de retina somados às retinografias totalizam 100% em cada categoria; † diferenças com significância (p<0,05); ‡ exceto pós-operatório tardio de facectomia sem intercorrências; § com possível acometimento do segmento posterior ocular, exceto diabetes e hipertensão arterial sistêmica; ¶ paciente que apresenta visão útil em apenas um dos olhos; || antecedente pessoal ou familiar, queixa ou quadro clínico compatíveis com doença ocular com possível acometimento do segmento posterior, exceto diagnóstico ou investigação de glaucoma primário de ângulo aberto ou alta miopia; # facectomia ou capsulotomia; ** uso de medicação oral com potencial toxicidade retiniana; †† diagnóstico ou investigação de glaucoma primário de ângulo aberto.

O estudo foi aprovado pelo Comitê de Ética da instituição, sob o número 2.338.968, CAAE: 77215417. 0.0000.0071.

Para análise da indicação da estratégia do exame, considerou-se que as seguintes situações eram elegíveis para avaliação por RET, em casos de boa acuidade visual (visão melhor ou igual a 20/30 no pior olho)^10^ e na ausência de sintomas (rastreamento): pacientes portadores de *diabetes mellitus* ; portadores de hipertensão arterial sistêmica; em uso de medicação oral com potencial toxicidade retiniana; com diagnóstico ou suspeita de GPAA; e com retinopatias estáveis e assintomáticas, exceto miopia maior que -3,00 dioptrias.^11^ Tais critérios não fazem parte de consenso e foram propostos para a realização deste estudo.

Em relação à análise do resultado do exame, a avaliação levou em conta ambos os olhos de cada paciente; quando havia mais de um diagnóstico, considerou-se a principal alteração retiniana − aquela que trazia maior risco à visão. Além do diagnóstico principal, avaliou-se também individualmente a presença de RD e do aumento da escavação papilar.

Para a análise estatística, foi utilizado o software IBM *Statistical Package for the Social Sciences* (SPSS), para Windows, versão 20 (IBM Corp, Armonk, NY, EUA). As variáveis contínuas estão descritas na forma de média e desvio padrão, e as variáveis categóricas, de frequências absolutas e relativas. Para comparar variáveis contínuas, foi utilizado o teste *t* de Student não pareado, e o teste χ_2_foi empregado na comparação de variáveis categóricas. O valor de p<0,05 foi considerado estatisticamente significativo.

## RESULTADOS

Foram avaliados 1.729 pacientes; destes, 1.190 realizaram MR e 539 realizaram RET. A figura 1 mostra o número total de pacientes que realizaram cada exame e apresentaram dados clínicos completos para análise. A média de idade dos pacientes foi 63,5±15,5 anos, e 66% eram do sexo feminino. O diagnóstico de diabetes estava presente em 32,2% dos pacientes.

**Figura 1:** Pacientes e modalidades de exame

Do total de 1.729 pacientes, foram excluídos 10 (0,6%; 9 que realizaram MR e 1 RET) nos quais não foi possível avaliar a indicação do exame. Não tinham indicação aparente para a realização do exame de retina conforme os critérios enumerados acima 58 pacientes (3,4%); 42 pacientes foram submetidos ao MR e 16 foram submetidos à RET. Na tabela 1 também estão expostos os motivos da indicação do exame nos casos em que o resultado foi normal, o que ocorreu em 166 pacientes (9,6% do total), sendo 115 MR (9,6% do total de MR) e 51 RET (9,5% do total de RET).

Em relação à modalidade de exame, 386 pacientes (22,4%) apresentavam indicação para exame de retina e eram elegíveis para a modalidade RET. De todos os pacientes elegíveis para RET, 229 (59,3%) foram submetidos ao MR, o que correspondeu a 13,3% do total geral. Os motivos para a solicitação de exames neste grupo de pacientes foram acompanhamento de retinopatias estáveis (DMRI, neurite óptica e nevo de coroide) em 16 pacientes (6,9%); uso de medicação oral com potencial de toxicidade retiniana em 8 pacientes (3,5%); diagnóstico de diabetes em 83 pacientes (36,2%); diagnóstico ou investigação de GPAA em 86 pacientes (37,5%); diagnóstico de hipertensão arterial sistêmica em 36 pacientes (15,7%).

Os principais diagnósticos encontrados nos exames de retina estão expostos na tabela 2 , sendo os mais frequentes o aumento da escavação papilar (30,7%) e a RD (13,2%). Na tabela 2 também estão enumerados os resultados do grupo de 58 pacientes que tiveram exames solicitados sem indicação aparente. Em 121 pacientes (115 MR e 6 RET), ou 7% do total de exames, a avaliação da retina detectou alterações periféricas nas formas de degeneração periférica, rotura ou buraco retiniano periférico, ou, ainda, descolamento de retina. Em 18 pacientes (1%), a avaliação foi impossibilitada em função de opacidade de meios (10, ou 0,8%, de MR e 8, ou 1,5%, RET).

**Tabela 2:** Principais diagnósticos, distribuições por modalidades de exame e distribuição dos exames solicitados sem indicação aparente

Diagnósticos	Total	Mapeamentos de retina	Retinografias	Exames sem indicação aparente
	n (%)	(%)	(%)	n (%)
Aumento da escavação papilar	401 (23,2)	20,3	29,5	2 (3,4)
Alterações periféricas	99 (5,7)	8,2	0,4	7 (12,1)
DMRI	110 (6,4)	5,9	7,4	4 (6,9)
Descolamento de retina	25 (1,4)	1,8	0,7	0 (0)
Distrofias retinianas	34 (2)	1,3	3,3	2 (3,4)
Impossível	18 (1)	0,8	1,5	0 (0)
Maculopatias tóxicas	8 (0,5)	0,7	0	1 (1,7)
Miopia degenerativa	93 (5,4)	6,6	2,8	0 (0)
Nevo de coroide	45 (2,6)	2,4	3,2	1 (1,7)
Normal	170 (9,8)	9,9	9,6	22 (37,9)
Oclusão vascular retiniana	55 (3,2)	3,1	3,3	1 (1,7)
Retinopatia diabética	221 (12,8)	13,1	12,1	0 (0)
Retinopatia hipertensiva	122 (7,1)	8,5	3,9	2 (3,4)
Miscelânea^*^	275 (15,9)	17,3	12,8	15 (25,8)
Sem dados	53 (3,1)	0,2	9,5	1 (1,7)
Total	1.729 (100)	100	100	58 (100)

^*^Inclui: alterações não glaucomatosas do nervo óptico, alterações pigmentares não associadas a distrofias, buraco de mácula, alterações congênitas, outras maculopatias não classificadas nos itens acima, outras doenças vasculares retinianas não classificadas nos itens acima, membrana epirretiniana, uveíte posterior, alteração do reflexo foveal, dobras de coroide, drusas de coroide não associadas à degeneração macular relacionada à idade, descolamento do vítreo posterior, fibras de mielina, hialose asteroide, *phthisis bulbi* , pós-operatório tardio de cirurgia vitreorretiniana, retinosquise degenerativa. DMRI: degeneração macular relacionada à idade.

No presente estudo, as principais indicações para a realização do exame de retina para pacientes avaliados em Unidade Básica de Saúde foram diabetes (23,7%) e investigação de glaucoma (23,5%); nos portadores de diabetes, o exame estava normal em 34,3% das vezes. Os principais diagnósticos foram aumento da escavação papilar (23,2%) e RD (12,8%); naqueles pacientes em que o resultado do exame foi normal, a principal indicação do exame foi a presença de diabetes (34,3%). Considerou-se que em apenas 3,4% dos casos o exame foi solicitado sem indicação aparente. O MR foi o exame mais realizado (68,8%); no entanto, considerando todos os pacientes que eram elegíveis para a realização da modalidade RET, aproximadamente 60% destes foram submetidos ao MR.

## DISCUSSÃO

Atualmente, não há consenso quanto à necessidade da realização do exame de retina sob midríase em indivíduos que não apresentem sinais ou sintomas de doenças do segmento posterior ocular, ou que não tenham fatores de risco para o desenvolvimento de tais doenças, já que condições detectadas em exame de rotina sob dilatação pupilar e que geram mudança de conduta ou prevenção de desfecho são raras.^11 - 13^ Como exemplos, podem-se citar a detecção de nevo de coroide como achado de exame e seu baixo risco de transformação maligna^14^ e alterações na periferia da retina não acompanhadas de sintomas, que geralmente são benignas e não demandam tratamento.^15^ No presente estudo, 121 pacientes (17%) apresentaram alterações retinianas periféricas (115 submetidos ao MR e 6 à RET).

A *American Academy of Ophthalmology* (AAO) recomenda que um exame oftalmológico completo de base seja realizado em pessoas com 40 anos, mas não menciona especificamente a dilatação pupilar.^16^ O Conselho Brasileiro de Oftalmologia (CBO) elaborou protocolo que recomenda a realização de MR nas seguintes situações: pré-operatório de cirurgia de catarata ou de cirurgia refrativa; entopsias agudas ou crônicas; altas miopias; antecedentes familiares ou pessoais de descolamento de retina, trauma ocular e retinopatias hipóxicas (diabetes, tromboses, doença de Eales e anemia falciforme).^17^

Entretanto, na prática clínica, exames para avaliação da retina são solicitados como parte da investigação oftalmológica ou clínica complementar em diversas outras condições oculares ou sistêmicas que potencialmente afetam o segmento posterior do olho. Considerando as duas principais estratégias de exame da retina, o MR e a RET, não parece haver indicações claras quanto às situações em que uma ou outra estratégia está mais indicada; a escolha deve levar em conta fatores como a disponibilidade do exame, a relação custo-efetividade e a situação clínica específica. Por exemplo: a investigação de lesões predisponentes a descolamento de retina em pacientes com miopia^18 , 19^ ou em pacientes com sintomas de descolamento do vítreo posterior é indicação clara de MR, que permite avaliar a extrema periferia da retina, ao contrário da RET convencional.^13^ Tal discussão é válida se considerarmos apenas os retinógrafos convencionais, pois já há aparelhos de campo ultra-amplo que permitem avaliar a extrema periferia da retina; no entanto, atualmente, seu custo é muito alto, o que impede seu uso presente no sistema público de saúde. Futuramente, com a popularização de tais equipamentos, é possível que esta discussão se torne anacrônica.^20^

Na maior parte das indicações do exame, não houve diferença significativa entre a solicitação de uma ou outra modalidade diagnóstica, com as seguintes exceções: miopia maior que -3,00 dioptrias, investigação de baixa acuidade visual e hipertensão arterial, em que predominou significativamente a indicação de MR; e a investigação de glaucoma, em que predominou significativamente a indicação de RET. Tampouco houve diferenças importantes nas distribuições de diagnósticos obtidos por cada modalidade.

Geralmente, os dois métodos diagnósticos para avaliação da retina estudados trazem resultados equivalentes e são estratégias intercambiáveis, com algumas situações em que a RET pode ser considerada o método de escolha em função do perfil custo-efetividade,^4 - 8 , 20^ já que é compatível com telemedicina e prescinde do especialista no momento do exame, havendo um custo elevado inicial para aquisição de equipamentos e montagem da estrutura, os quais tendem a se diluir posteriormente. Na situação estudada, o serviço já contava com os aparelhos, o que conferia a esta série um melhor perfil de custo-efetividade para a estratégia da RET. Em nosso meio, estudos avaliaram a adequação da RET associada à telemedicina para o alívio da demanda,^8 , 18 , 21^ principalmente no rastreamento da RD em um cenário de epidemia global de diabetes.^22^ No atual estudo, as RET foram realizadas sob midríase e atingiram uma boa qualidade técnica, sendo que apenas 1,5% das RET não permitiram leitura por má qualidade da imagem.

Adicionalmente, a avaliação por RET digital permite a interação com algoritmos de inteligência artificial, para o auxílio no rastreamento e na tomada de decisões clínicas, o que se apresenta como tendência para futuro.^23^ Além disso, o sistema atual de obtenção das imagens, baseado em aparelhos de grande porte, deve passar por grande transformação, com a possibilidade de obtenção de imagens a partir de retinógrafos portáteis ou smartphones.^24^

Durante a preparação deste artigo, o Brasil encontrava-se em meio a polêmica sobre a padronização da telemedicina, a partir de resolução do Conselho Federal de Medicina publicada em fevereiro de 2019 e revogada no dia 22 do mesmo mês (resolução 2.227/2018).^25^ Deve-se ressaltar que, se, por um lado, o emprego da telemedicina pode representar a ampliação do acesso ao diagnóstico, por outro lado, o exame de retina é apenas uma parte do exame oftalmológico presencial, que inclui, ainda, anamnese, exame de refração, biomicroscopia, motilidade ocular extrínseca e tonometria.

Entre os pontos fortes deste estudo, podemos ressaltar o grande número de pacientes examinados em um mesmo centro ao longo de um período de aproximadamente 10 anos, e a avaliação de um dos cenários que corresponde à realidade em nosso meio. A apresentação destes dados pode auxiliar no planejamento de protocolos que objetivam melhor emprego de recursos. Entre as limitações do estudo, mencionamos o possível viés da escolha do exame devido a restrições de agenda, além de pacientes que tiveram o exame solicitado e não chegaram a realizá-lo. Outro viés diz respeito à documentação fotográfica de pacientes com diagnóstico ou suspeita de glaucoma, já que tinham a disponibilidade de um tipo especial de RET, a estéreo-fotografia de papila, que documenta o nervo óptico com maior aumento. Muitos pacientes desta categoria podem ter sido submetidos a esta modalidade, desta forma subestimando o diagnóstico de glaucoma ou suspeita de glaucoma na amostra estudada. Mesmo assim, o diagnóstico ou a suspeita de glaucoma foram duas das principais indicações de exames, e o aumento da escavação papilar foi o principal diagnóstico encontrado. Finalmente, há casos de pacientes que tiveram ambas as modalidades de exames solicitadas, mas apenas o primeiro exame a ser realizado foi considerado para a análise de indicações e resultados, o que constitui mais um viés em nossos resultados e conclusões.

## CONCLUSÃO

Os dados avaliados neste estudo apontaram que as principais indicações para solicitação do exame de retina foram o diabetes e a investigação de glaucoma, e os principais resultados dos exames foram o aumento da escavação papilar e a retinopatia diabética. Na ausência de diretrizes bem definidas para a solicitação de exames para a avaliação da retina, e sem critérios para a escolha da modalidade de exame da retina, os dados revelaram que, na prática diária, a modalidade de exame é escolhida geralmente de maneira intercambiável. Considerando que as diferentes estratégias estudadas possuem perfis distintos de custo-efetividade, sugere-se a criação de diretrizes quanto às indicações para a realização de exames de retina e a padronização da modalidade mais indicada em cada situação, de modo a otimizar recursos do sistema público de saúde.
